# Stretchable, Bio-Compatible, Antioxidant and Self-Powering Adhesives from Soluble Silk Fibroin and Vegetal Polyphenols Exfoliated Graphite

**DOI:** 10.3390/nano11092352

**Published:** 2021-09-10

**Authors:** Luca Valentini, Maria Rachele Ceccarini, Raquel Verdejo, Gianluca Tondi, Tommaso Beccari

**Affiliations:** 1 Civil & Environmental Engineering Department, Università degli Studi di Perugia and INSTM Research Unit, Strada di Pentima 4, 05100 Terni, Italy; 2 Department of Pharmaceutical Sciences, University of Perugia, 06123 Perugia, Italy; mariarachele.ceccarini@unipg.it (M.R.C.); tommaso.beccari@unipg.it (T.B.); 3 Department of Polymeric Nanomaterials and Biomaterials, Institute of Polymer Science and Technology, ICTP-CSIC, 28006 Madrid, Spain; r.verdejo@csic.es; 4 Department of Land Environment Agriculture and Forestry, University of Padua, 35020 Legnaro, Italy; gianluca.tondi@unipd.it

**Keywords:** regenerated silk, exfoliated graphite, tannin extracts, mechanical properties, adhesion, biocompatibility

## Abstract

The development of bio-glues is still a challenging task, regarding adhesion on wet surfaces; often, high performance and adaption to complex geometries need to be combined in one material. Here, we report biocompatible adhesives obtained by blending regenerated silk (RS) with a soluble plant-derived polyphenol (i.e., chestnut tannin) that was also used to exfoliate graphite to obtain graphene-based RS/tannin (G-RS/T) composites. The resultant G-RS/T hybrid material exhibited outstanding stretchability (i.e., 400%) and high shear strength (i.e., 180 kPa), superior to that of commercial bio-glues, and showed sealant properties for tissue approximation. Moreover, we showed how such nanocomposites exhibit electromechanical properties that could potentially be used for the realization of green and eco-friendly piezoelectric devices. Finally, we demonstrate the in vitro glue’s biocompatibility and anti-oxidant properties that enable their utilization in clinical applications.

## 1. Introduction

Adhesives that can bond biological tissues are of great interest in the areas of wound repair, applications in surgical sutures and regenerative medicine [1,2,3]. To date, there are some commercial adhesives for diverse wet surfaces, but which, however, have limits in terms of excessive mechanical rigidity or poor mechanical toughness [4,5]. A multifunctional adhesive that overcomes the mismatch between planar geometries and the curved interfaces of most natural objects (i.e., living organisms and environmental substrates), is sticky on wet substrates and has self-sensing properties for real-time monitoring of vital parameters needs a huge effort for implementation. 

Regenerated silk (RS), due to its biocompatibility and biodegradation properties, has been extensively investigated in wound dressings [6] and scaffolds [7,8]. Moreover, RS has already been studied as a bio-ink for printable smart architectures [9] as well as a colloidal-type glue when blended with polyethylene glycol (PEG), polyvinyl alcohol or tannic acid [10,11,12]; however, these hybrid materials suffer from a long gelation time.

Tannins are polyphenol extracts produced by superior plants as a chemical shield to protect the wooden structure against radical oxidative processes, as well as against biologic attacks from animals, fungi and bacteria [13]. These extracts are widely applied in the leather tannery row, in pharmacy and cosmetics, but also in oenology and animal feeding, suggesting high compatibility with living tissue [14]. Flavonoid tannins have recently found application for polymeric application in adhesives [15], wood preservatives [16], bio-plastics [17] and foams [18] thanks to their excellent capacity to crosslink with a contained amount of hardener (e.g., formaldehyde, hexamine, furanics).

One underexploited but highly interesting property of polyphenols lies in their capacity to exfoliate graphene [19]. Graphene is an ideal additive for adhesives because its structure involves significant enhancements on the mechanical properties of the composite. This is of particular interest when applied on regenerated silk [9].

Recently, it was reported how two-dimensional (2D) nanomaterials, thanks to their outstanding physical and electrical properties, may play a crucial role when interfaced with elastomeric-like matrices to fabricate flexible smart devices [20]. Combining high-performance 2D material, such as graphene, with biogenic materials (i.e., RS and tannin) is a versatile strategy to develop smart materials. The materials were chosen on the basis of the following criteria: the adhesive has to be compliant to the dynamic movements of tissues and self-sensing when the interface is stressed. 

Here, we propose a reverse engineering approach to fabricate stretchable and self-powering materials, exploiting the tannin-assisted exfoliation of graphite nanoplatelets in the same solvent used to regenerate silk from silk cocoons. The synergistic effect of the addition of graphene and tannin endows the resultant composites with remarkably improved mechanical properties and electromechanical properties, as well as adhesion ability on wet surfaces, suggesting an exciting material platform of nature-based sealants, which could be used for a wide range of applications in regenerative medicine.

## 2. Materials and Methods

### 2.1. Materials

Silk cocoons were supplied by a local farm (Fimo srl, Milano, Italy). Sodium hydrogen carbonate (NaHCO_3_), calcium chloride (CaCl_2_) and formic acid (FA) were supplied by Merck (Darmstadt, Germany). Graphite nanoplatelets C777 (GNPs, carbon content > 65%, average flake thickness ~ 20 nm, layer number ~ 41, average particle (lateral) size:16 μm, were supplied by Nanesa (Arezzo, Italy). Commercial chestnut tannin extract “Saviotan A” was kindly supplied by SavioLife (Viadana, Italy).

### 2.2. Synthesis of the Films

Silk cocoons were degummed with NaHCO_3_ (5 g in 200 mL of water) in boiling water for 30 min and rinsed with deionized water; the procedure was repeated two times. The degummed fibers were then left to dry at room temperature.

RS solutions were produced by dispersing the degummed silk fibers into the FA/CaCl_2_ solution by magnetic stirring at room temperature for 5 min to obtain a homogeneous solution; the CaCl_2_ amount was calculated with respect to the silk:CaCl_2_ weight ratio (i.e., 60:40) with a silk amount of 0.65 g that was dissolved in FA (5 mL). Afterwards, 1 wt% and 10 wt% of tannin, with respect to the silk content, were added to the solution, respectively, leaving the dissolution to proceed at 50 °C. Finally, 1 wt% of GNPs, with respect to the silk amount, was added to the solutions and sonicated for 30 min at room temperature. The solutions were dropcast into Petri dishes with a diameter of 5 cm and left to evaporate under a laminar flow hood overnight. Hereinafter, RS/T is used for samples obtained by adding only tannin, and G-RS/T for those obtained by adding GNPs (Table 1). For comparison purposes, we also prepared films of neat RS.

### 2.3. Characterization

To investigate the exfoliation of GNPs, 1 wt% of GNPs powder, calculated with respect to the RS amount, was added to 5 mL FA/CaCl_2_ solutions containing 0 wt%, 1 wt% and 10 wt% of tannin, respectively. The GNPs powder was dissolved with bath sonication operating at 100 W and 40 kHz for 30 min. The dispersions were then left to stand for 24 h to allow the insoluble graphite to precipitate, and were then centrifuged for 30 min at 500 rpm.

FTIR investigations were carried out on the casted films using a PerkinElmer (Waltham, MA, USA) spectrometer equipped with a diamond crystal in attenuated total reflectance (ATR) mode. 

The sheets were removed from the Petri dishes using a sharp razor blade and were placed onto the diamond ATR crystal before applying pressure to the top of the sample. Measurements were performed in the range of 4000 to 400 cm^−1^ with a resolution of 4 cm^−1^, and the number of scans was 64 for each spectrum. The measurements were performed at room temperature. The diamond crystal was cleaned after each measurement and background spectra were acquired prior to each sample measurement. The spectra were collected in the range of 1750 to 1520 cm^−1^, which are the amide I and amide II bands of RS. The spectra were then deconvoluted, firstly by smoothing the signal with a polynomial function—in this case a 15-point Savitski–Golay smoothing function—then by subtracting a linear baseline and applying Gaussian deconvoluting curves by Origin 9 (OriginPro, version 9.0, OriginLab Corporation, MA, USA) software.

The morphology of the exfoliated graphite was investigated by atomic force microscopy (AFM) and the extent of exfoliation was analyzed with Raman spectroscopy. AFM images were obtained using a tapping mode AFM (multimode Nanoscope Iva, Digital Instrument/Veeco Aschheim, Germany) under ambient conditions. The oscillation frequency was set to approximately 300 kHz. The GNP suspensions were deposited by spin-casting onto previously clean Si wafers. The height and length of the GNP particles were measured for at least 20 particles. The Raman spectra were recorded in a Renishaw Invia (Renishaw plc., Wotton-under-Edge, UK) microscope using an excitation wavelength of 514.5 nm argon ion laser and 0.02 cm^−1^ resolution. 

The stress–strain curves of the RS/T and G-RS/T films (3 cm × 1.5 cm rectangular shaped samples) were obtained through a tensile testing machine (Lloyd Instr. Ltd. LR30K, Steyning Way West Sussex, UK). The samples were tested at room temperature with a strain rate of 5 mm∙min^−1^ using a 50 N load cell. Three samples per formulation were tested. 

The adhesive properties were tested by gluing two portions of commercial porcine intestine (supplied by Butcher’s Falchi, Terni, Italy) to the prepared samples. Once the materials were applied between porcine intestines, the structures were stored in a climatic chamber for 24 h at 37 °C and a relative humidity of 90%. The shear strength was then calculated with a strain rate of 5 mm·min^−1^, dividing the maximum force by the adhesion area. The rupture was observed on the overlapped region of the intestine. To avoid damage of the tissues by the clamping, polystyrene stiff films (NIST^®^ SRM^®^ 1921b, Merck, Milan, Italy) were glued to the back of the intestine as the substrate. The accurate values of the adhesion areas were calculated by importing the digital photos in AutoCAD (AutoCAD LT, version F.51.0.0, Autodesk Inc., CA, USA, 2012). The reported results are the average of at least three measurements per composition of adhesive.

The sealant properties of the prepared samples were determined measuring the burst pressure. A 5 mm-long incision was made through one porcine intestinal wall. RS/T and G-RS/T sealants were positioned and gently pressed over the incision. A Porcine intestine loop was sectioned with a scalpel. The two loop cuts created were then anastomosed with 5/0 absorbable interrupted full thickness sutures; the suture used was synthetic absorbable monofilament made from the polyester (p-dioxanone) [21]. A digital manometer was connected in series to a pump, recording the burst pressure, which is defined as the maximum pressure reached before its sharp loss after the rupture. The piezoelectric properties were investigated as follows: the RS/T and G-RS/T samples were stuck onto the bottom of an aluminum tape electrode, and then aluminum tape was attached to the top. The samples were then positioned over a Teflon substrate to electrically isolate the system. The samples were progressively stretched and the open circuit voltage values were monitored using a computer-controlled Keithley 4200 Source Meter Unit (Tektronix UK Ltd., The Capitol Building, Oldbury, UK). The dynamic piezoelectric output of the samples was measured by using the finger imparting method reported elsewhere [22].

### 2.4. Antioxidant Assay by Oxygen Radical Absorbance Capacity (ORAC)

The antioxidant capacity of all compounds was determined using the ORAC method. Hydrophilic extraction was performed on two independent occasions for each sample. The procedure was based on the method of Zulueta et al. [23], with slight modifications [24]. Briefly, 2,20-azobis (2-methylpropionamide) dihydrochloride (AAPH) was used as a peroxyl radical generator, fluorescein was used as a fluorescent probe and Trolox was used as a reference antioxidant standard. The data are expressed as micromoles of Trolox equivalents (TE) per gram of sample (μmol TE/g). The ORAC assay was carried out on a FLUOstar OPTIMA microplate fluorescence reader (BMG LABTECH, Offenburg, Germany) at an excitation wavelength of 485 nm and an emission wavelength of 520 nm.

### 2.5. Cytotoxicity Assay In Vitro

For the bio-compatibility assessment, the HepG2 cell line (ATCC HB 8065), a human hepatocyte carcinoma, was used as a representative model. This cell line was purchased from ATCC (American Type Culture Collection, Gaithersburg, MD, USA). According to ATCC standard procedure, HepG2 was cultured in Eagle’s minimum essential medium (EMEM), supplemented with 10% heat-inactivated fetal bovine serum (FBS), 1% non-essential amino acids, 1 mM sodium pyruvate, 2 mM of L-glutamine and antibiotics (100 U/mL penicillin, 100 μg/mL streptomycin) [25]. To evaluate the biocompatibility of these compounds, MTT assay was used after 24 h and 48 h of treatment [26]. HepG2 cells were seeded in a 96-well plate at 1 × 10^4^ cells/well final cell density and after 24 h the fresh complete medium was replaced with 8 different scalar dilutions of stock solutions (1 mg/mL). MTT reagent (0.5 μg/μL) was added in each well for 3 h at 37 °C. Then, the supernatant was carefully removed, and formazan salt crystals were dissolved in 100 μL DMSO 100%, and the OD values were measured spectrophotometrically at 570 nm (Eliza MAT 2000, DRG Instruments GmbH, Marburg, Germany). Each experiment was performed in triplicate three times and cell viability was expressed as a percentage relative, as previously described [27,28]. A one-way ANOVA test was performed using the Graphpad program (GraphPad Prism 9.2.0.332, GraphPad software, San Diego, CA, USA) for MTT assay.

## 3. Results and Discussion

**Figure 1:**
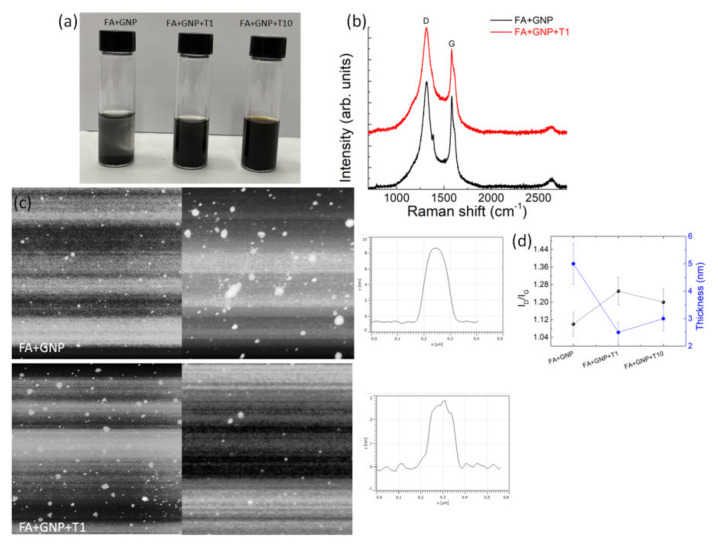
(**a**) Photographs of FA/CaCl_2_ dispersions of GNPs with concentrations of 0 (FA+GNP), 1 (FA+GNP+T1) and 10 (FA+GNP+T10) wt% of tannin, respectively. (**b**) Raman spectra of FA+GNP and FA+GNP+T1 dispersions. (**c**) AFM images (5 µm × 5 µm on the left and 3 µm × 3 µm on the right) showing the topographic view and height profiles of GNPs on silicon obtained from FA+GNP (top) and FA+GNP+T1 (bottom) dispersions after centrifugation, respectively. (**d**) ID/IG ratio values and flake thickness obtained from Raman and AFM analyses on the prepared solutions.

By exploiting the experimental evidence that formic acid (FA) is able to solubilize degummed silk and that tannins are also generally soluble in acidic environments [9,14], we investigated the ability of FA solution of CaCl_2_ mixed with tannin to exfoliate the graphite. Figure 1a shows that the addition of tannin to the FA+GNPs dispersion after 10,000 rpm centrifugation for 20 min forms stable brown solutions, suggesting that tannin exfoliates graphite in FA. Raman and AFM analyses were used to support the exfoliation process of graphite. Figure 1b shows the Raman spectra of the graphite dispersed in FA (i.e., FA+GNP) and 1 wt% tannin-assisted dispersion of GNPs in FA (i.e., FA+GNP+T1). The features of graphite are the G band at 1580 cm^−1^, the D band at 1324 cm^−1^ and the 2D band at 2700 cm^−1^. After the addition of tannin, the band at 1324 cm^−1^ increased its intensity. The D band indicates the presence of defects while the G band represents sp^2^ hybridization in-plane stretching vibration of carbon atoms [29]. Thus, the D band intensity can be used to explore the number of defects of the graphene layers due to the graphite exfoliation [30]. According to the Raman spectra (Figure 1b), the D band is increasing in intensity because the presence of tannin exfoliates graphene, increasing the ID/IG ratio from 1.10 to 1.25. An ID/IG ratio of 1.20 was found for the 10 wt% tannin-assisted dispersion of GNPs in FA (i.e., FA+GNP+T10, Figure S1, Supplementary Materials). AFM analysis was performed to investigate the thickness of the graphite layers. Figure 1c shows graphite flakes that are exfoliated by the addition of the tannin; i.e., the height of the flakes obtained from the FA+GNP+T1 solution was found to be 2.5 nm, against a mean value of about 8 nm measured for the flakes of the FA+GNP dispersion. When compared with the raw graphite dispersed in FA, these findings suggest that most of the graphite was exfoliated by using FA/tannin solution under sonication (Figure 1d).

**Figure 2:**
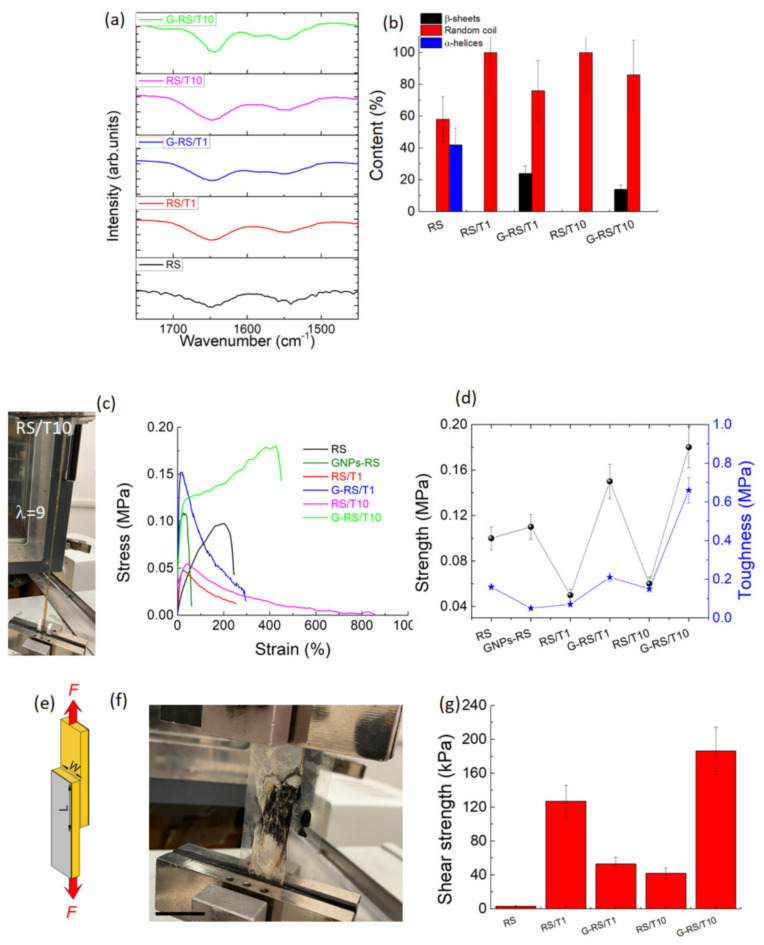
(**a**) FTIR spectra in amide I and amide II regions of RS, RS/T and G-RS/T samples obtained by the addition of 0 wt%, 1 wt% (RS/T1) and 10 wt% (RS/T10) of tannin to the RS and by the addition of GNPs (G-RS/T1 and G-RS/T10), respectively. Peak deconvolution carried out by peak assignment to the secondary structures: 1650 cm^−1^ (random coil), 1656 cm^−1^ (α-helices) and 1620 cm^−1^ (β-sheet) for amide I and 1540 cm^−1^ (random coil) for amide II. (**b**) Quantitative analysis of secondary structures in RS/T and G-RS/T samples, respectively. (**c**) Photograph (left) demonstrating excellent elongation of RS/T10 film when stretched and (right) stress/strain curves for the RS/T and G-RS/T samples. (**d**) Tensile strength and toughness of the prepared specimens calculated from the engineering stress–strain curves. (**e**) Setup for measurement of shear strength (F, force; L, length; W, width). (**f**) Photographs show three G-RS/T10 films adhered on porcine intestines. The scale bar indicates 1.5 cm. (**g**) Shear strength values between porcine intestine of RS/T and G-RS/T adhesives after storing the samples at 37 °C and relative humidity of 90%.

Structural changes in the RS, depending on the tannin content and GNPs addition, were investigated by FTIR spectroscopy. The spectra reported in Figure 2a and Figure S2 show the amide I and amide II regions, where the absorptions between 1622–1637 and 1697–1703 cm^−1^ correspond to β-sheet structure, and those at 1638–1655 cm^−1^ and 1656–1662 cm^−1^ are indicative of random-coil and α-helical structure, respectively [31]. The ratio of the peak area with respect to the total area was used to estimate the percentage content of secondary structures that are summarized in Figure 2b. According to previous results [9,32], neat RS consists of random coil and α-helices structures while, for the G-RS/T1 and G-RS/T10 samples, part of the random coils is transformed in β-sheets structures (Figure 2b). It is noteworthy that tannins interact with proteins, producing stable complexes; therefore, the presence of only random coils in the RS/T samples can be ascribed to the presence of polyphenols that negatively affect the β-sheet and/or α-helices arrangement. The β-sheet content observed in the G-RS/T composites is due to a uniform distribution of the SF chains on the graphene surfaces, inferring a better interfacial interaction and a higher tensile strength of the composite film [33].

For evaluating the practicability of the RS/T and G-RS/T composites as stretchable materials, the mechanical properties were evaluated in detail (Figure 2c). The synergistic effect of the addition of both tannin and graphene for the RS composite is illustrated in Figure 2d. As a result, the mechanical properties of the G-RS/T10 sample are improved, with the highest tensile strength of 0.18 MPa, toughness (calculated as the area under the engineering stress-strain curve) of 0.66 MPa and elongation at break of 430%, respectively. Additionally, the mechanical properties of the RS added with different tannin concentrations (i.e., 1 wt% and 10 wt%) were also investigated and indicate that tannin addition also has a large effect on the mechanical performance. Compared with neat RS, we observed a decrease in the tensile strength and an increase in the elongation at break. These mechanical properties can be explained as follows: the molecules of tannin hinder the secondary bonding between the protein chains (intra- and inter-molecular), establishing a stable complex through non-covalent bonds [34]. This new arrangement facilitates the sliding of the molecules so that the tensile strength reduces while the elongation increases.

To evaluate the adhesion performance of the RS/T and G-RS/T materials, we measured the shear strength by lap-shear test. Considering the wide utilization of soluble plant-derived polyphenols in the cosmetic industry [35], we tested the adhesion performance of the prepared samples on wet porcine skin, a model tissue very similar in its mechanical properties to human skin [35] (Figure 2e,f and Figure S3).

The G-RS/T10 composite can establish a strong (i.e., 180 kPa) adhesion between wet porcine skins upon the application of gentle pressure (1 kPa) for a few seconds (Figure 2g). The adhered porcine skins exhibit a similar strength even more than 48 h after the specimen preparation. These findings demonstrate superior adhesion properties compared with commercially available glues (Dermabond ≈ 40 kPa, BioGlue ≈ 50 kPa, Coseal ≈ 5 kPa and Tisseel ≈ 2 kPa) [36,37]. Considering the shear strength recorded for the RS/T1 glue (i.e., 120 kPa), we can claim that both the tannin-assisted graphite exfoliation and the subsequent dispersion in soluble RS are necessary for the adhesion of wet surfaces. In particular, it can be noticed that the shear strength of RS/T1 is higher because tannins create stable complexes with the proteins of silk, creating a tighter adhesive. However, if tannin is added in excess, the polymer becomes too rigid and the composite breaks soon. When graphite nanoparticles are added, the tannin is mainly sequestered to facilitate exfoliation and it does not participate in the adhesion process. Considering these phenomena, the outstanding results registered for G-RS/T10 can be explained by the fact that tannin is enough for exfoliating graphene, and the excess contributes to the tightening effect of the adhesive.

**Figure 3:**
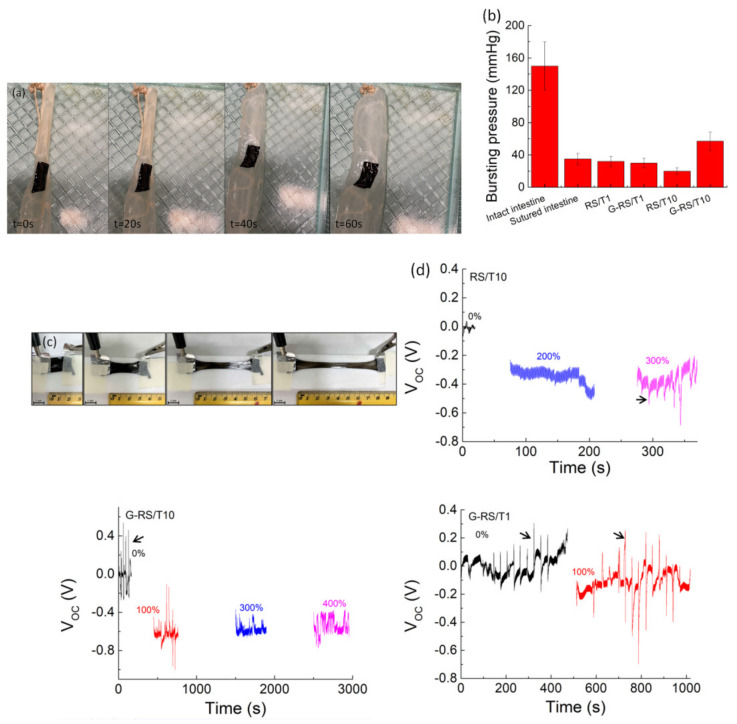
(**a**) Procedure of the ex vivo porcine intestine burst pressure test for RS/T and G-RS/T patches. Incision of the intestine segment and application of the patch over the incision and testing at different times till rupture was observed (Movie S1). (**b**) Porcine intestine burst pressures on the incised intestine. The burst pressures of intact intestine and sutured intestine are also reported for comparison purposes. (**c**) Photographs showing the configuration for the piezoelectric effect measurement with G-RS/T10 sample as the active component and its elongation. (**d**) The generated open-circuit voltage as a function of the engineering strain of RS/T and G-RS/T samples, respectively. The arrows indicate the open circuit voltage recorded using the finger imparting method.

We exploit the properties of such materials in potential applications, investigating as a proof-of-concept a sealing patch of a perforated porcine intestine (with a 0.5 cm wide hole) (Figure 3a and Supplementary Movie S1). The closure of wounds from surgical operations is a very critical aspect to reduce hospitalization times, avoid tissue perforation and reduce inflammatory phenomena [38,39,40]. The resistance of the sealing patch made with the prepared samples was evaluated by measuring the bursting pressure (Figure 3b). Intestinal anastomosis with mechanical sutures withstands low pressure before bursting (i.e., 35 mmHg) in accordance with our previous observations [21]. The use of the G-RS/T10 glue applied on incised intestine increases the burst pressure to 57 mmHg, an increment of 60% in comparison to the value recorded for sutured intestine. 

As RS is a piezoelectric bio-polymer [41], we enable the utilization of RS/T and G-RS/T composites as smart patches able to behave as self-powering strain sensors (Figure 3c). Stretchable RS/T and G-RS/T hybrid sensors were prepared by putting a conductive Al adhesive on the top and bottom sides of the samples (Figure 3c). Such a setup could serve as a versatile platform for implantable devices to adhere onto wet and complex structures. The results reported in Figure 3d suggest that voltage generation can be activated by stretching the samples. Beyond the voltage output increasing with mechanical strain, we superimposed a dynamic mechanical test by applying a constant load every 30 s and the resulting electrical output signal was characterized (indicated by the arrows in Figure 3d). The output voltage rises and decays with the application of a force, envisaging its potential for force measurement. With consideration of the outputs generated from different RS/T and G-RS/T samples, the device with the G-RS/T10 adhesive shows on average the largest difference between the maximum (press) and minimum (release) values. In general, we observe that the RS/T performance can be further increased in terms of the output voltage by adding GNPs. Here, the combined effect of tannin on (i) exfoliating GNPs and (ii) randomizing RS arrangement involves an enhancement of the contact between GNPs chains during elongation with consequent increases in voltage.

**Figure 4:**
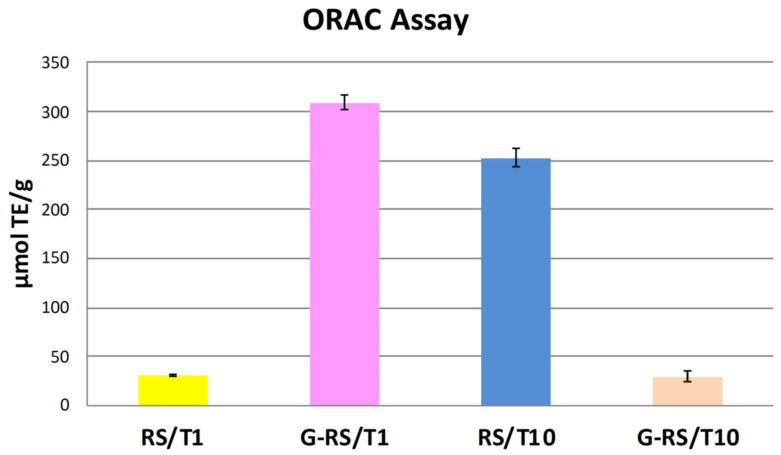
ORAC assay (for all compounds). Results are expressed as mean ± SD of two independent extractions, each one performed in triplicate.

Envisioning a biomedical application, antioxidant capacity was initially investigated due to the tannin presence in both the RS/T compounds (1 wt% or 10 wt%) and in the G-RS/T composites. The tannin extract alone shows, as expected, a great antioxidant property: 20,268.25 ± 521.55 μmolTE/g (data not shown). The RS/T1 compound, reported in Figure 4, has approximately 30 μmolTE/g, whereas RS/T10 exhibits an ORAC value about eight times greater, i.e., 252 μmolTE/g. We ascribe this result to a higher tannin content. The most interesting result is the antioxidant capacity of G-RS/T1, which displays the highest ORAC value of all four compounds: 308 μmolTE/g. It is likely that the antioxidant activity is amplified by the presence of graphene [42,43]. On the contrary, the low ORAC value obtained for G-RS/T10 is due to the partial insoluble nature of this compound in the hydrophilic buffer, which could underestimate the result.

**Figure 5:**
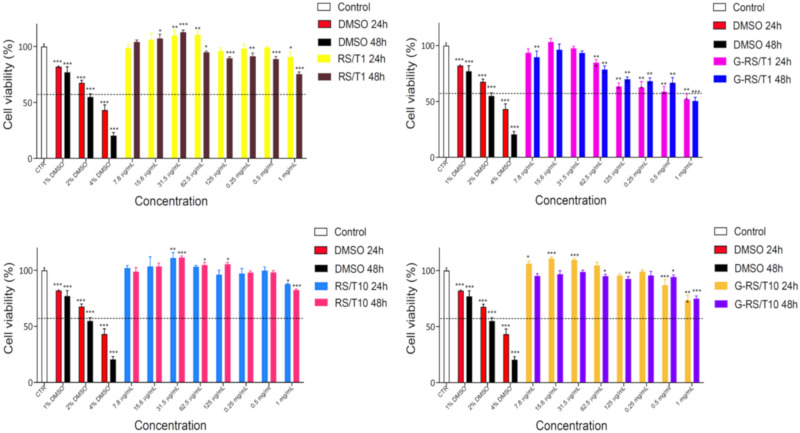
Safety evaluation of the RS/T and G-RS/T samples tested in vitro using MTT test. HepG2 cells were in vitro treated with six scalar concentrations for 24 h and 48 h. The percentage of viable cells with respect to the control was reported as the mean standard deviation of three independent experiments, each conducted in triplicate. Positive controls were obtained with DMSO 1%, 2% and 4%. Values were represented as mean ± standard deviation and were compared with one-way ANOVA with *p* ≤ 0.05 considered statistically significant; * *p* < 0.01, ** *p* < 0.001 and *** *p* < 0.0001.

MTT assay was used to investigate cell viability at different concentrations of four compounds after 24 h and 48 h of treatment. The lower concentrations tested (i.e., 7.8, 15.6, 31.5 and 62.5 μg/mL) are almost safe for the HepG2 cell line, as shown in Figure 5. In some cases, these compounds are able to increase viability, probably due to the antioxidant property of tannin. RS/T1 and RS/T10 are cytotoxic only at the highest concentration used (i.e., 1 mg/mL), whereas the G-RS/T1 shows a reduced cell viability starting from 125 μg/mL. A possible explanation of this result is ascribable to the presence of exfoliate graphene and to the high intrinsic antioxidant capacity, which has the power to become pro-oxidant in a dose-dependent manner. The G-RS/T10 exhibits the same problem of lacking solubility already observed during ORAC assay, and for this reason the results are not completely trustworthy.

## 4. Conclusions

In this paper, we report a method to exfoliate graphite nano-platelets in FA-soluble plant-derived polyphenol. This method allows us to produce tough regenerated silk composite films and adhesive properties on wet and complex substrate geometries. The presence of exfoliated graphite in the RS/T allows an enhanced piezoelectric conductivity. These materials couple their sealant and piezoelectric properties, offering advantages over existing tissue adhesives by enhancing the performance and allowing electric monitoring of the strain. Finally, after proving their excellent antioxidant properties and their biocompatibility for contained concentration (<125 µg/mL), these materials also provide new opportunities for engineering bio-materials in regenerative medicine. The synergic effect observed by the combination of these compounds of natural origin represents a breakthrough in polymer science in line with the global circular economy concept.

## Figures and Tables

**Table 1:** Composition of the prepared samples.

Sample	Tannin (wt%)	GNPs (wt%)
RS	0	0
RS/T1	1	0
G-RS/T1	1	1
RS/T10	10	0
G-RS/T10	10	1

## Data Availability

No data are available.

## Supplementary Materials

The following are available online at https://www.mdpi.com/article/10.3390/nano11092352/s1. Figure S1: Raman spectrum of G-RS/T10 sample; Figure S2: Deconvolution of FTIR transmittance spectra, amide I region, of RS/T and G-RS/T samples; Figure S3: Force vs. displacement of the prepared samples for the shear strength measurement; Movie S1.
