# Supplementary material for: Stretchable, Bio-Compatible, Antioxidant and Self-Powering Adhesives from Soluble Silk Fibroin and Vegetal Polyphenols Exfoliated Graphite
Source: Nanomaterials (Basel). 2021 Sep 10;11(9):2352. doi: 10.3390/nano11092352 (PMC8472279; doi:10.3390/nano11092352)
Supplement: Supplementary file 1 [file nanomaterials-11-02352-s001.zip › nanomaterials-1342781-supplementary.pdf]

Supplementary Materials

Stretchable, Bio-Compatible, Antioxidant and Self-Powering Adhesives from Soluble Silk Fibroin and Vegetal Polyphenols Exfoliated Graphite

Luca Valentini [1,*], Maria Rachele Ceccarini [2], Raquel Verdejo [3], Gianluca Tondi [4] and Tommaso Beccari [2]

[1] Civil & Environmental Engineering Department, Università degli Studi di Perugia and INSTM Research Unit, Strada di Pentima 4, 05100 Terni, Italy
[2] Department of Pharmaceutical Sciences, University of Perugia, 06123 Perugia, Italy; mariarachele.ceccarini@unipg.it (M.R.C.); tommaso.beccari@unipg.it (T.B.)
[3] Department of Polymeric Nanomaterials and Biomaterials, Institute of Polymer Science and Technology, ICTP-CSIC, 28006 Madrid, Spain; r.verdejo@csic.es (R.V.)
[4] Department of Land Environment Agriculture and Forestry, University of Padua, 35020 Legnaro, Italy; gianluca.tondi@unipd.it (G.T.)
* Correspondence: luca.valentini@unipg.it (L.V.); Tel.: +39-0744-492924

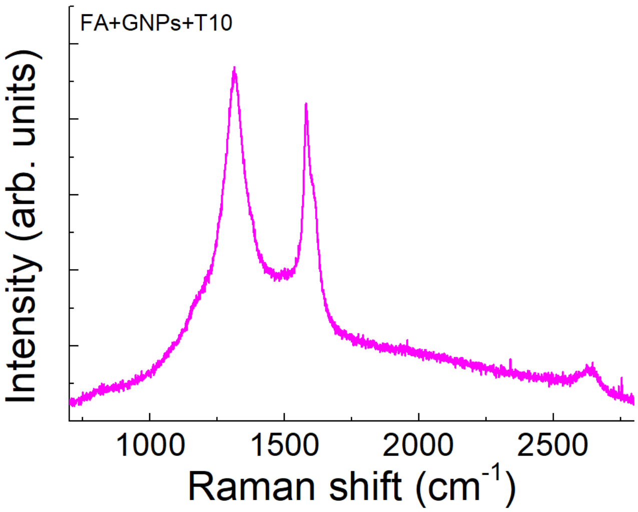

Figure S1. Raman spectrum of G-RS/T10 sample.

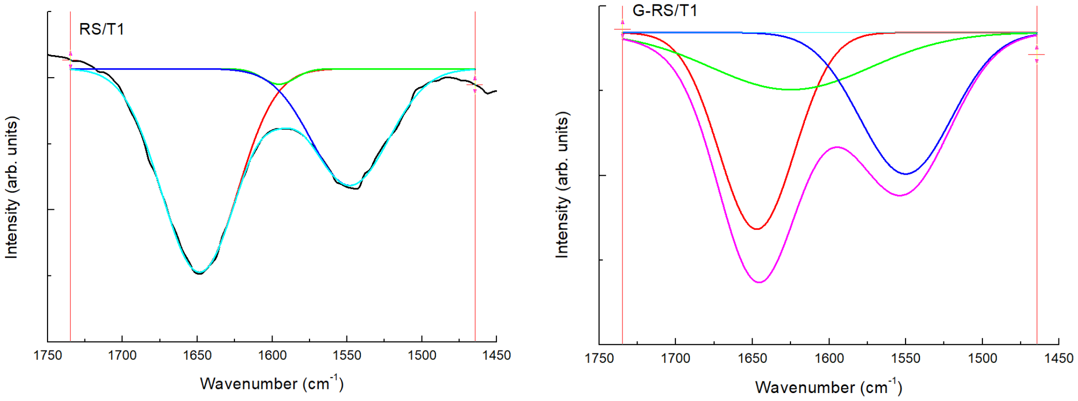

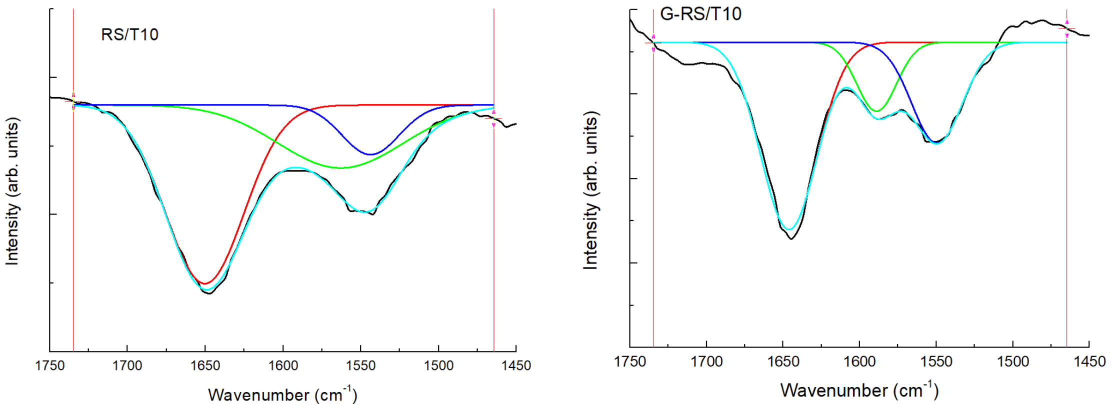

Figure S2. Deconvolution of FTIR transmittance spectra, amide I region, of RS/T and G-RS/T samples.

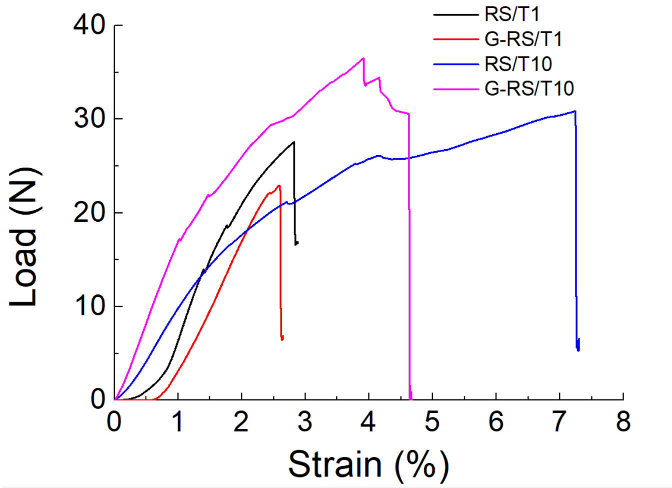

Figure S3. Force vs. displacement of the prepared samples for the shear strength measurement.
